# Cell deconvolution-based integrated time-series network of whole blood transcriptome reveals systemic antiviral activities and cell-specific immunological changes against PRRSV infection

**DOI:** 10.1186/s13567-025-01451-w

**Published:** 2025-01-22

**Authors:** Byeonghwi Lim, Chiwoong Lim, Min-Jae Jang, Young-Jun Seo, Do-Young Kim, Christopher K. Tuggle, Kyu-Sang Lim, Jun-Mo Kim

**Affiliations:** 1 Functional Genomics & Bioinformatics Laboratory, Department of Animal Science and Technology, Chung-Ang University, Anseong, Gyeonggi-do 17546 Republic of Korea; 2 Department of Animal Science, Iowa State University, Ames, IA 50011 USA; 3 Department of Animal Resources Science, Kongju National University, Yesan, Chungcheongnam-do 32439 Republic of Korea

**Keywords:** PRRS virus, whole blood, RNA sequencing, cell deconvolution, transcriptomics, time-series network

## Abstract

Porcine reproductive and respiratory syndrome (PRRS) causes significant economic losses in the swine industry. However, the molecular mechanisms behind the common and cell type-specific systemic responses during PRRS virus (PRRSV) infection are not well understood. In this study, we collected viremia data, antibody levels, and whole-blood RNA-seq data obtained from eight PRRSV-infected piglets. We utilised a cell deconvolution approach to calculate cell type enrichment, constructed a time-serial gene co-expression network with differentially expressed genes, and conducted functional annotations. Three significant modules were identified within the network. The changes associated with viremia revealed an upregulated expression of genes related to antiviral activity. In the T-cell- and NK-cell-specific modules, infection led to an increased T-cell population and upregulation of genes related to T-cell defence responses. Conversely, in the monocyte- and neutrophil-specific module, genes involved in inflammatory responses were downregulated due to a decrease in monocyte proportion. This study highlights the time-series antiviral activities associated with viremia and the transcriptomic changes associated with immune responses in specific cell types. The findings provide comprehensive insights into host responses to PRRSV infection, including diagnostic biomarkers.

**Supplementary Information:**

The online version contains supplementary material available at 10.1186/s13567-025-01451-w.

## Introduction

Porcine reproductive and respiratory syndrome (PRRS) is an infectious viral disease that causes reproductive failures in breeding sows and respiratory problems in growing pigs [1, 2]. It is caused by the PRRS virus (PRRSV), a single-stranded RNA virus, and is considered the most critical disease affecting commercial pig production globally [3].

PRRSV specifically targets porcine alveolar macrophages (PAM). The infection results in the destruction of these cells due to the virus’s rapid replication. This interaction harms the host’s innate immune response, as demonstrated by the limited production of antiviral cytokines, namely interferons α and β (IFN-α/β) [4, 5].

Examining the main target organs affected by PRRSV is essential for understanding the fundamental mechanisms of the disease. However, studies involving blood samples studies are also crucial because they minimise unnecessary testing and enhance our understanding understanding of the host’s systemic immune response, thereby complementing tissue studies. As a result, blood analysis is a vital component of PRRSV research [6, 7].

For instance, a previous study focused on verifying the presence of quantitative trait loci associated with resistance to PRRSV (NVSL 97–7985 strain) in blood samples. This research aimed to explore the whole blood transcriptomic differences in weaning pigs based on their WUR genotype [8, 9]. Additionally, researchers identified different gene expression signatures in pregnant gilts associated with low or high foetal mortality rates following PRRSV (NVSL 97-7985 strain) infection, through whole blood RNA-seq [10].

Despite these efforts, there is still a limited number of studies focusing on the comprehensive transcriptome analysis of whole blood, specifically in relation to the PRRSV JA142 strain.

Whole blood is composed of various cells, including immune cells such as monocytes, neutrophils, T-cells, B-cells, and NK-cells, all of which contribute to the host’s innate and adaptive immune responses [11]. Therefore, to understand the immunological changes in PRRSV-infected pigs, it is essential to monitor the alterations in the cellular composition and function of their blood.

Cell deconvolution is an emergent method that uses bulk RNA-seq data to estimate the proportions of different cell types in samples. This technique offers valuable insights into cell types and has the potential to enhance our understanding of inter- and intra-cellular dynamics and the state of various cellular subsets during PRRSV infection.

This study employed a time-sequential experimental design to identify systemic changes in the mechanisms affected by PRRSV infection and to uncover modifications in cell-type-specific functions.

## Materials and methods

### Experimental animals with detection of viremia and antibodies

**Figure 1:**
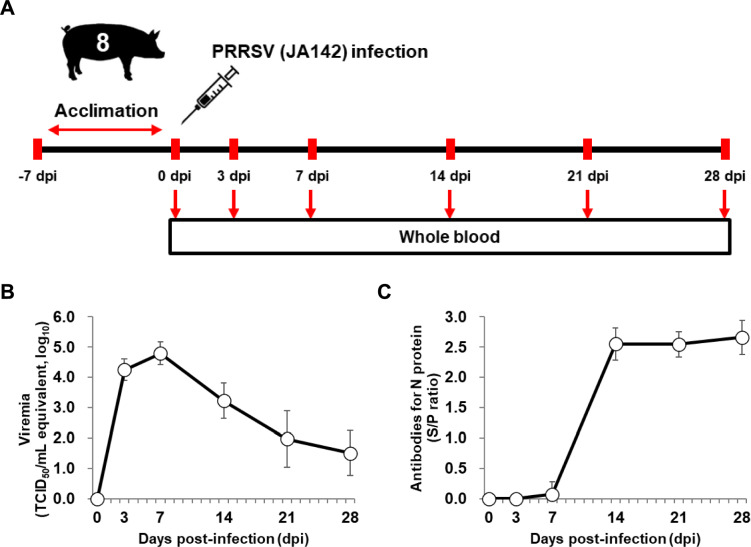
**Overview of the experimental design and phenotypes**. **A** Schematic representation of the PRRSV research. Whole blood samples were obtained at 0, 3, 7, 14, 21, and 28 days post-infection. **B** Viremia levels in the PRRSV-infected piglets. **C** Levels of antibodies in the PRRSV-infected piglets.

Four-week-old piglets (Landrace × Yorkshire × Duroc; *n* = 8) were sourced from a PRRSV-negative farm and housed in the animal facilities at our institution (Figure 1A). After a seven-day acclimatisation period, the eight piglets were challenged intramuscularly with 2 mL of PRRSV (1 × 10^3^ tissue culture infectious dose (TCID)_50_/mL), which was diluted in sterile PBS. The PRRSV-2 strain JA142 (GenBank: AY424271.1) was utilised in this study. Whole blood was collected from pigs at 0, 3, 7, 14, 21, and 28 days post-infection (dpi), and the serum was separated to detect viremia and PRRSV antibodies, as outlined in a previous study [12].

The Jeonbuk National University Institutional Animal Care and Use Committee, Republic of Korea, approved all animal experiments (approval number 2016–43).

### RNA extraction, complementary DNA (cDNA) library construction, and RNA-Seq

Whole blood samples were collected from the piglets using Tempus Blood RNA Tubes (Thermo Fisher Scientific, Waltham, MA, USA) through standard venipuncture. Immediately after collection, the tubes were shaken vigorously for 10 s to ensure thorough mixing of the blood with the RNA-stabilising solution, according to the manufacturer’s instructions. For long-term storage, the samples were kept at −20 °C. The stabilised blood was then transferred to a 50-mL tube and diluted with 1 × PBS for RNA extraction, which was performed according to the manufacturer’s instructions (Tempus Spin RNA Isolation Kit User Guide, Thermo Fisher Scientific). We obtained 45 high-quality RNA samples out of a total of 48 samples.

Following the manufacturer's protocols, the extracted RNA was used for library preparation with the TruSeq Stranded Total RNA LT Sample Prep Kit (Human Mouse Rat) (Illumina, San Diego, CA, USA). The prepared libraries were quantified using a Qubit 2.0 Fluorometer (Life Technologies, Carlsbad, CA, USA) and validated with an Agilent 2100 Bioanalyzer (Agilent Technologies, Santa Clara, CA, USA) to confirm the insert size and calculate molar concentration. The indexed libraries were analysed using an Illumina HiSeq4000 instrument (Illumina), and paired-end (2 × 100 bp) sequencing was performed.

### Data preprocessing

To select the appropriate quality-filtering strategy, we conducted a quality check of the raw read data for each sample using FastQC software v0.11.7. Based on the quality results, we then trimmed the reads with adaptors using Trimmomatic software v0.38. After trimming, we re-evaluated the reads with FastQC and mapped them to the reference genome (Sus scrofa 11.1.95, GCA_000003025.6) of the Ensembl genome browser using the default options of the HISAT2 v2.1.0 program.

Raw gene counts for each library were calculated using the exons in the Sus scrofa GTF v95 (Ensembl) as the genomic annotation reference file, utilising the featureCounts tool from the Subread package v1.6.3. We excluded the haemoglobin subunit alpha (*HBA*) and haemoglobin subunit beta (*HBB*) genes from the analysis. Out of 45 samples, we retained 43 samples with library sizes exceeding 10 million after quality control.

The analysis focused on 10737 genes that were expressed in at least four samples, with counts per million (CPM) greater than one.

### Differentially expressed gene (DEG) analysis

Normalised and scaled expression values derived from RNA-seq data were analysed using a generalised mixed model (GMM) to account for potential nuisance factors [13]. The model included fixed effects for dpi to capture temporal changes, while individual piglets were included as random effects to address individual-level variation. The RNA integrity number (RIN) was also included as a covariate to control for variability associated with RNA quality.

Least-squares means were calculated to represent the average expression levels across different dpi. DEGs were identified for all time points relative to the baseline (0 dpi) using stringent criteria: a false discovery rate (FDR) of less than 0.05 and an absolute log_2_ fold change (FC) of at least 1. Gene expression patterns were visualised using volcano plots to illustrate the number of DEGs at each dpi. Bar plots summarised the upregulated and downregulated genes, providing a clear visual representation of changes in expression direction.

An overlapping analysis was conducted to identify differentially expressed genes across multiple time points, with overlapping DEGs for each dpi visually represented using a Venn diagram. Additionally, multidimensional scaling (MDS) was performed using the R package “limma” to demonstrate the similarity among samples based on their gene expression patterns.

### Cell deconvolution analysis

Cell deconvolution was conducted using filtered gene expression data consisting of 10 737 genes, following human-based gene annotation across all samples with the xCell method [14]. Only the predicted cell types that were significant (*P* < 0.2) in more than half of the samples (≥ 22), were selected for further network analysis.

### Network construction and functional analyses

Weighted gene co-expression network analysis (WGCNA) was employed to identify co-occurring modules among genes and phenotypes, including predicted cell types, to better understand their relationships. A signed network method was utilised in this analysis. The thresholding power was calculated to selectively prune branches from the dendrogram based on the geometry of the clusters. The adjacency matrix was then transformed into a topological overlap matrix (TOM) to minimise false connections during module identification. The gene expression patterns for each module are expressed as log_2_FC values at each dpi. Functional enrichment analysis was conducted for each module by integrating Kyoto Encyclopaedia of Genes and Genomes (KEGG) pathways using the Database for Annotation, Visualization, and Integrated Discovery (DAVID) v6.8. Additionally, biological processes (BPs) were analysed using gene ontology (GO) terms, applying stringent criteria (*P* < 0.05 and counts ≥ 5) along with the DIRECT option for GO annotation filtering. The enriched GO terms were visualised using treemaps created with the REVIGO tool. KEGG annotations were also enriched using the same cut-off criteria. All data used in the enrichment analyses were annotated to *S. scrofa*.

### Gene modulation

Pathview was utilised to analyse the cytokine-cytokine receptor pathway, which was consistently enriched across the three identified modules, to assess gene modulation. The mean log_2_FC values of the DEGs within each module were presented together. Based on the log2FC values, a heatmap was created to visualise the variations in gene expression at each dpi.

## Results

### PRRSV viremia and antibodies

The viremia kinetics exhibited a notable temporal pattern throughout the infection period. A sharp increase was recorded at 3 dpi, where a significant spike was observed. The peak levels of viremia occurred at 7 dpi, followed by a subsequent decline (Figure 1B). In contrast, antibodies for the N protein remained stable at 0, 3, and 7 dpi, with a notable increase observed at 14 dpi (Figure 1C). These distinct patterns highlight 3 and 7 dpi as critical time points for viremia peaks, while 14 dpi seems to spark a significant rise in antibodies targeting the N protein epitopes.

### Transcriptomic data processing and DEG profiling

RNA-seq data were collected at 0, 3, 7, 14, 21, and 28 dpi with PRRSV for each of the eight individuals. The raw reads average was 40,912,962, and the post-trimming average was 38,921,067. The unique mapping rates averaged 73.44%, and the overall mapping rates averaged 93.93% (Additional file 1).

**Figure 2:**
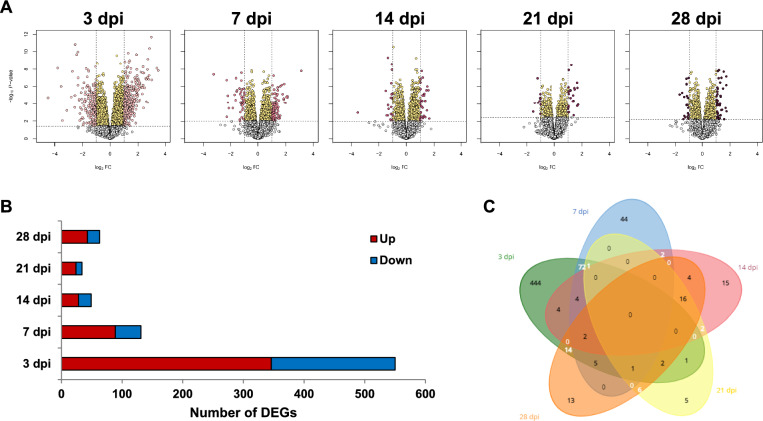
**Time-serial transcriptome profiling in whole blood during PRRSV infection**. **A** Time-serial volcano plots indicating differentially expressed genes (DEGs) were calculated using 0 days post-infection (dpi) as a reference. Significance was determined with FDR < 0.05 and absolute log_2_FC ≥ 1. **B** The number of DEGs at different time points (3, 7, 14, 21, and 28 dpi). **C** The Venn diagram shows overlapping DEGs.

After mapping the reads to the porcine reference genome, visualisation using an MDS plot revealed significant differences between the individuals (Additional file 2). The number of DEGs, compared to expression levels at 0 dpi, displayed distinct trends: the highest number of DEGs (550) was observed at 3 dpi, followed by a decrease to 131 DEGs at 7 dpi, 49 DEGs at 14 dpi, and 34 DEGs at 21 dpi. A notable increase in the number of DEGs occurred at 28 dpi, with 63 DEGs identified (Figure 2A). These results underscored the dynamic and evolving nature of the gene expression response to the infection.

Analysis of DEGs showed a consistent pattern in upregulated and downregulated expressions compared to the reference at 0 dpi. Specifically, the number of DEGs with upregulated expression (*n* = 410) greatly exceeded those with downregulated expression (*n* = 249) (Figure 2B, Additional file 3). The immune response induced by PRRSV infection is expected to manifest as systemic responses through the bloodstream, suggesting there are more active signals than inhibitory ones. The number of common DEGs across each time point is illustrated in Figure 2C.

### Cell type enrichment

Cell deconvolution analysis, utilising transcriptomic data, allowed for the estimation of raw enrichment scores for each cell type in the individuals studied. This comprehensive analysis included 64 distinct cell types. The predicted cell proportions were determined after estimating the raw enrichment scores for each cell type for all individuals (Additional file 4). Significant scores for cell types in each individual were identified using a significance threshold of 0.2 (Additional file 5). Out of the 64 cell-type enrichment scores, 29 were found to be significant. Changes in raw enrichment scores for six representative cell types (monocyte, neutrophil, CD8^+^ T-cell, NK cell, erythrocyte, and platelet) are presented in Additional file 6.

### Gene co-expression network (GCN)

To identify the different co-expression modules following PRRSV infection in each phenotype (dpi, viremia, antibody, and enrichment scores for specific cell types), we conducted a WGCNA based on the pairwise correlation of gene expression for 680 genes. At least one gene showed significance in five comparisons. Five modules (turquoise, green, yellow, blue, and brown) exhibited significant correlations with the phenotype (Additional file 7).

The turquoise module (216 genes) strongly correlated with monocyte—and neutrophil-specific cell types. The green module (36 genes) correlated with T cells, while the yellow module (75 genes) correlated with both T and NK cells. The blue module (197 genes) was linked to erythrocyte—and platelet-specific types, and the brown module (101 genes) correlated with viremia-specific types (Additional file 8).

All modules were found to be associated with only a small number of specific cell types. In contrast, a previous study suggested that various cell types contribute to the overall response [9]. During PRRSV infection, changes in the transcriptome of whole blood may be influenced by multiple cells, primarily white blood cells. Nevertheless, the association with specific cells appears to be stronger in this study due to the stringent criteria used to select significant genes for network construction.

**Figure 3:**
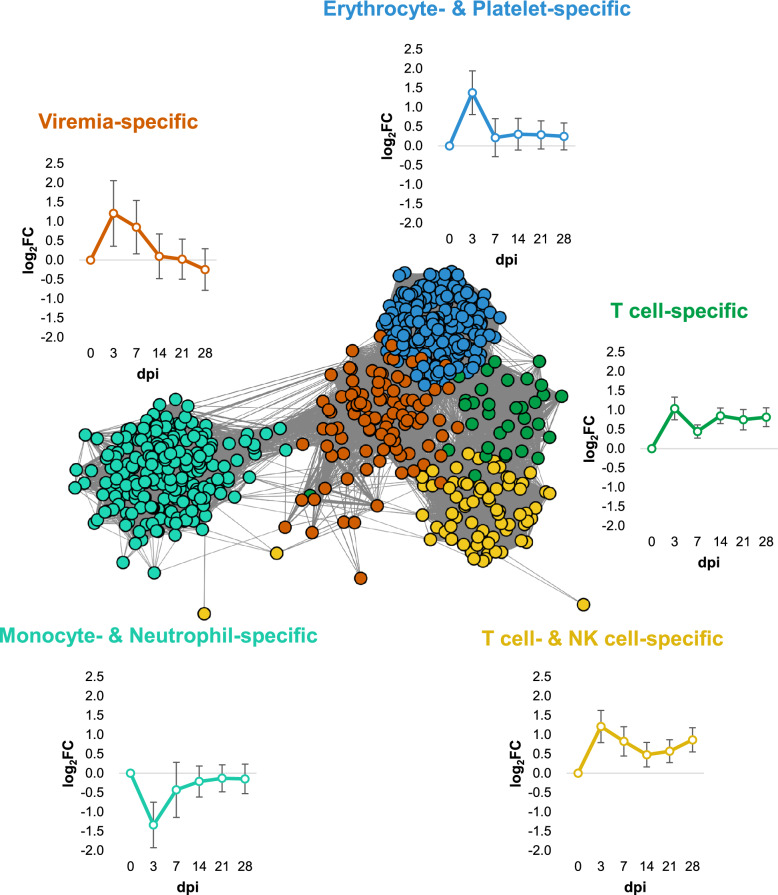
**Gene co-expression network (GCN) combined with cell deconvolution in whole blood during PRRSV infection**. GCN, consisting of 623 nodes (genes) and 44327 edges (interactions), was constructed using WGCNA. The colour of each node is separated according to clustered modules: viremia-specific (brown), T cell- and NK cell-specific (yellow), monocyte- and neutrophil-specific (turquoise), erythrocyte- and platelet-specific (blue), and T cell-specific (green). Expression patterns in each module were visualised around the GCN.

Based on the WGCNA results, we constructed a GCN with 623 genes and 44327 significant connections (Figure 3). The nodes within the network were clustered into distinct modules. Notably, genes in the brown (viremia-specific) and blue (erythrocyte- and platelet-specific) modules exhibited a significant increase in expression at 3 dpi, followed by a subsequent decrease. The genes in the green (T-cell-specific) module also showed an initial increase at 3 dpi, followed by a decrease at 7 dpi, but then experienced another increase at 14 dpi. The yellow (T- and NK-cell-specific) module increased at 3 dpi, followed by a decrease and then another rise at 21 dpi. In contrast, the turquoise (monocyte- and neutrophil-specific) module was initially downregulated at 3 dpi, followed by an increase in gene expression.

### Functional annotations

**Figure 4:**
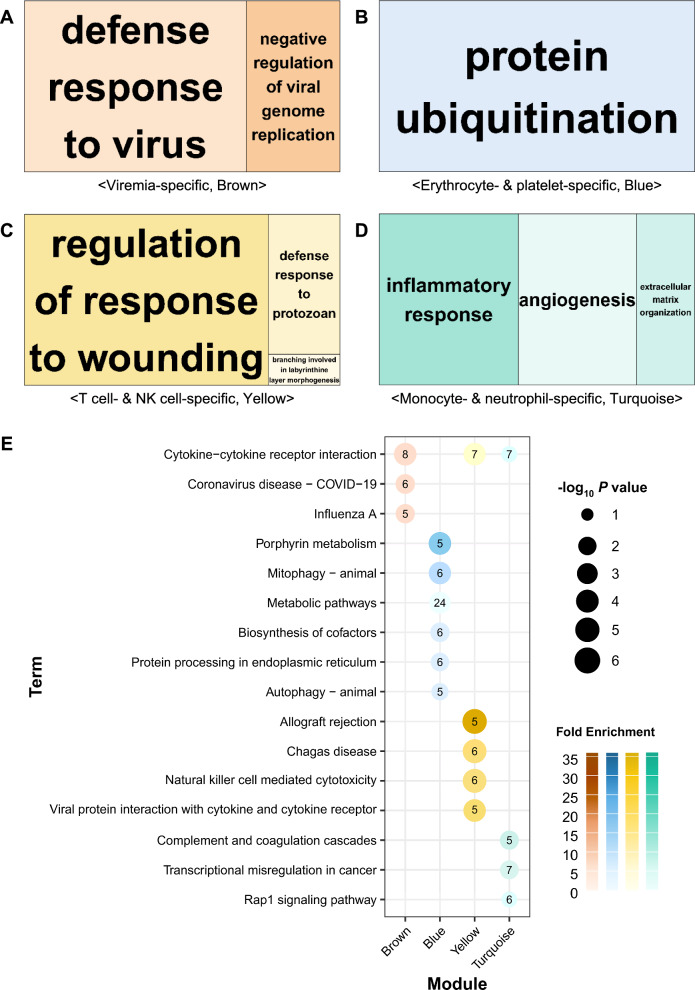
**Functional enrichments representing biological processes in each module**. **A** Viremia-specific, **B** Erythrocyte- and platelet-specific, **C** T-cell- and NK-cell-specific, and **D** Monocyte- and neutrophil-specific treemaps enriched in biological processes during Gene Ontology (GO) analyses. **E** Kyoto Encyclopedia of Genes and Genomes (KEGG) enrichment in each module.

GO enrichment analyses were conducted to identify the BPs of DEGs in each module (Additional file 9). The results are illustrated in treemaps, with −log_10_ *P*-values represented as area sizes (Figures 4A–D). The analyses revealed that certain GO terms were enriched in “defence response to virus” in the viremia-specific (brown) module, “protein ubiquitination” in the erythrocyte- and platelet-specific (blue) module, “regulation of response to wounding” in the T-cell- and NK-cell-specific (yellow) module, and “inflammatory response” in the monocyte- and neutrophil-specific (turquoise) module.

Additionally, we determined the enriched pathways for the DEGs using the KEGG database (Figure 4E; Additional file 9), which yielded results consistent with the GO terms analyses. The viremia-specific module included pathways such as “cytokine-cytokine receptor interaction”, “coronavirus disease—COVID-19”, and “influenza A”. The erythrocyte- and platelet-specific module contained pathways related to “porphyrin metabolism”, “mitophagy—animal”, “biosynthesis of cofactors, protein processing in the endoplasmic reticulum”, and “autophagy—animal”.

In the T-cell- and NK-cell-specific modules, the enriched pathways included “allograft rejection”, “Chagas disease”, “natural killer cell-mediated cytotoxicity”, “viral protein interactions with cytokines and cytokine receptors”, and “cytokine-cytokine receptor interactions”. For the monocyte- and neutrophil-specific modules, the significant pathways included “complement and coagulation cascades”, “transcriptional misregulation in cancer”, “cytokine-cytokine receptor interaction”, and the “Rap1 signalling pathway”.

The analysis revealed significant enrichment patterns across the various modules. Notably, “cytokine-cytokine receptor interaction” was particularly enriched in the brown module, “porphyrin metabolism” in the blue module, “allograft rejection” in the yellow module, and “complement and coagulation cascades” in the turquoise module. We specifically focused on “cytokine-cytokine receptor interaction”, which was significantly enriched in the brown, yellow, and turquoise modules. Overall, the cell-type enrichments across all modules aligned with their respective functions.

**Figure 5:**
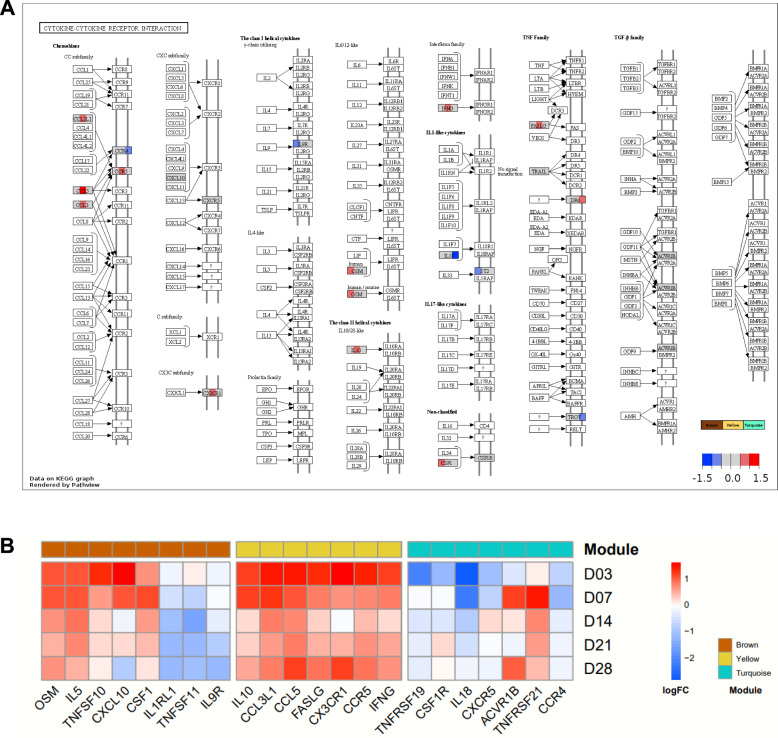
**Gene modulation in the cytokine-cytokine receptor interaction**. **A** Average log_2_ fold change (FC) values of differentially expressed genes (DEGs) throughout different time points (3, 7, 14, 21, and 28 days post-infection in each module: viremia-specific (brown; left), T cell- and NK cell-specific (yellow; middle), and monocyte- and neutrophil-specific (turquoise; right). **B** Heatmap indicating log_2_FC values of DEGs at each time point and module.

The study on gene modulation concerning “cytokine-cytokine receptor interaction” across the three identified modules (brown, yellow, and turquoise) revealed several linked genes. These included *OSM*, *IL5*, *TNFSF10*, *CXCL10*, *CSF1*, *IL1RL1*, *TNFSF11*, and *IL9R* in the brown module; *IL10*, *CCL3L1*, *CCL5*, *FASLG*, *CX3CR1*, *CCR5*, and *IFNG* in the yellow module; and *TNFRSF19*, *CSF1R*, *IL18*, *CXCR5*, *ACVR1B*, *TNFRSF21*, and *CCR4* in the turquoise module (Figure 5A). To visualise the expression levels of the enriched genes involved in cytokine-cytokine receptor interactions at each time point, a heatmap was generated for the three modules (Figure 5B).

## Discussion

Whole blood from pigs infected with the PRRSV (JA142 strain) was analysed to investigate systemic host responses through time-series transcriptomes, focusing on phenotypes that included the predicted proportions of cell types. Viremia and PRRSV-specific antibodies (N protein) were presented as previously described (Figures 1B and C) [12, 15–19]. The highest number of DEGs was identified at 3 dpi, with a sharp decline at 7 dpi and then a slight increase again at 28 dpi (Figure 2). This pattern differed from that observed in the lungs and bronchial lymph nodes (BLN), where the highest numbers of DEGs were found at 10 dpi after PRRSV infection [12]. These results suggest that systemic responses in the blood occur more rapidly than local responses in target organs, such as the lungs and lymph nodes.

Viral infections trigger the migration of circulating monocytes to the target organs, a process influenced by pro-inflammatory cytokines [20]. Additionally, the proportions of lymphocytes may increase or decrease depending on the specific virus involved [21, 22]. In the case of PRRSV infection, the enrichment scores for monocytes and neutrophils rapidly decreased in the early stage (3 dpi). During the same period, there was a slight increase in CD8^+^ T-cells (Additional file 6). Therefore, it is believed that changes in the proportion of blood cells following PRRSV infection can be indirectly assessed by analysing cell type enrichment.

Changes over time in the proportions of different cell types and viremia-specific responses were identified using an integrative network along with cell deconvolution (Figure 3). In the whole blood transcriptome analysis of the NVSL 97–7985 strain, a gene expression cluster associated with innate immunity, similar to that in the brown module (viremia-specific), showed an increasing expression pattern until 4 dpi, followed by a gradual decrease by 14 dpi [8, 9].

Additionally, a cluster akin to the turquoise module (specific to monocytes and neutrophils) revealed a decrease in gene expression at 4 dpi, which then gradually increased by 14 dpi. However, this cluster was enriched in cell signalling pathways rather than functions directly related to monocytes and neutrophils. In a separate study using the WUH3 strain, a gene cluster associated with both innate and humoral immunity was identified, which mirrored the expression pattern of the brown module (viremia-specific) and increased up to 11 dpi [23]. Moreover, a gene cluster related to cell cycle regulation displayed decreased expression at 4 dpi, followed by an increase of 11 dpi.

Overall, for the JA142 and NVSL 97-7985 strains, the immune response initially increased following infection but then declined at an early stage. In contrast, for the WUH3 strain, the immune response continued to grow over time, which is considered to be a hallmark of the highly pathogenic strain. This study focussed on constructing the network with a selected set of fewer genes based on strict criteria, which likely contributed to the observed differences and more specific results. When combined with the analysis of cell type enrichment, the changes in gene expression levels of the viremia-specific module reflect the overall immune response over time after PRRSV infection. Conversely, the alterations in gene expression levels of the other cell type-specific modules likely indicate changes in the proportions of specific cells within whole blood.

The viremia-specific (brown) module indicated a “defence response to virus” (Figure 4A) and showed enrichment of “influenza A” (Figure 4E, Additional file 9). These findings were consistent with the expression patterns observed in target organs (lung, BLN, and tonsil) at 3 dpi expression pattern of target organs in a previous study [12]. Notably, four genes (*IFIH1*, *CXCL10*, *MX1*, and *RSAD2*) in “influenza A” were found to be upregulated at 3 dpi, mirroring the expression patterns in the organs. This suggests that these genes could serve as valuable biomarkers for indicating indirect expression in local organs.

*IFIH1* encodes MDA5 (a member of the RIG-I-like receptor family) and is a cytoplasmic sensor of viral infections. CXCL10 has been shown to exhibit both protective and pathogenic functions in response to various viral infections, depending on the type of infection. However, its correlation with PRRSV remains unclear [24]. Both MxA (*MX1*) and viperin (*RSAD2*), possess interferon-inducible antiviral activities against RNA and DNA viruses [25], and it has been reported that they are regulated by lncRNAs during PRRSV infection [26].

Additionally, three genes (*OSM*, *IL5*, and *CSF1*) from “cytokine-cytokine receptor interactions” were found to be upregulated (Figure 5), particularly at 3 and 7 dpi. These genes activate the STAT3 protein (by *OSM* and *IL5*) and STAT5 protein (by *IL5* and *CSF1*) protein [27], which play crucial roles in the differentiation of specific T-cells, such as T helper 17 cells associated with STAT3 [28] and regulatory T-cells associated with STAT5 [29]. OSM is also recognised for enhancing the antiviral effects of IFN-α and acting as an inducer of adaptive immune responses during infections [30, 31]. Furthermore, OSM has been reported to inhibit PRRSV replication in MARC-145 cells [32].

These findings suggest that systemic antiviral activities increase in direct response to viremia in the bloodstream during PRRSV infection. The results also provide potential biomarker candidates for regulating host responses in target organs.

The cellular composition of blood changes in response to internal factors, such as ageing, and external factors like pathogen infections [33, 34].

Cell deconvolution in RNA-seq allows researchers to estimate the proportions of specific cell types and enrich their functions at the cellular level. This study confirmed functional enrichment and cytokine expression using two modules: T-cell- and NK-cell-specific, and monocyte- and neutrophil-specific (Figure 3). In the T-cell- and NK-cell-specific modules, gene expression related to innate and adaptive immune responses was significantly upregulated at 3 dpi. This expression then decreased at later time points but remained at a lower level of upregulation (Figures 3, 4C). Key genes in this module include interferon-γ (IFNG), which is produced by antigen-activated T-cells. IFNG stimulates adaptive antigen-specific responses and innate cell-mediated immune responses, primarily through macrophages (Figure 5) [35].

Additionally, CCL5, which is primarily expressed by T-cells and monocytes, is known to be induced by TNF-α and IFN-γ during pathogen infections, particularly viral infections [36, 37]. In pigs infected with PRRSV, CD8^+^ T-cells showed a significant increase in *CCL5* levels [38]. This indicates that the population of activated T-cells in the bloodstream rises to enhance defence responses against PRRSV infection in the early stages, with a sustained level of expression in relation to adaptive immunity.

In the monocyte- and neutrophil-specific modules, gene expression linked to inflammatory responses was observed to be rapidly downregulated at 3 dpi, followed by a gradual recovery (Figures 3 and 4D). Among the genes in this module, *IL18*, emerged as one of the most downregulated genes at both 3 and 7 dpi. This gene is known to be present in blood monocytes, including macrophages [39]. Additionally, *CSF1R* is also known to be highly expressed in monocytes and macrophages (Figure 5) [40].

These findings suggest that monocytes in the bloodstream may either migrate to local target organs or undergo early cell death following PRRSV infection. This observed decrease in inflammatory responses may be attributed to this reduction in the number of monocytes.

This study identified time-serial antiviral activities linked to viremia and immunological changes associated with specific cell types, namely monocytes and T-cells, by analysing whole blood transcriptomes using WGCNA and cell deconvolution. T-cells are known to increase in number and activate in response to PRRSV infection during the early stages of the infection, maintaining some presence even at later time points. In contrast, monocytes either migrate to the target organs or die early in the infection period. These findings provide a comprehensive overview of systemic host responses in whole blood and suggest potential biomarkers for diagnosis. By employing cell deconvolution, the analysis of whole blood transcriptomes reveals broad cellular changes and their associated functions, allowing for a more detailed understanding of cellular responses.

## Supplementary Information

**Additional file 1.** **Overview of data processing**.

**Additional file 2.** **Multidimensional scaling (MDS) based on whole blood transcriptomes in PRRSV infection.** Individual effects were found to be greater than temporal effects. These features were considered and adjusted while profiling the differentially expressed genes.

**Additional file 3.** **Detailed information of all differentially expressed genes**.

**Additional file 4.** **Raw enrichment scores in each cell type are estimated from cell deconvolution**.

**Additional file 5.** **Significance levels in each cell type are estimated from cell deconvolution**.

**Additional file 6.** **Changes in raw enrichment scores for representative cell types**.

**Additional file 7.** **Module-phenotype correlations were combined with cell-type enrichments generated by cell deconvolution**.

**Additional file 8.** **Detailed information of differentially expressed genes in each module**.

**Additional file 9.** **Functional enrichment in each module**.

## Data Availability

The raw read data used in this study have been deposited in the NCBI Sequence Read Archive (SRA) under accession no. PRJNA1039980.

## Abbreviations

BPs: biological processes
CPM: counts per million
DAVID: Database for Annotation, Visualization, and Integrated Discovery
dpi: days post-infection
FDR: false discovery rate
FC: fold change
GO: gene ontology
GMM: generalised mixed model
IFN-α/β: interferons α and β
IFNG: interferon-γ
KEGG: Kyoto Encyclopedia of Genes and Genomes
MDS: multidimensional scaling
PRRS: porcine reproductive and respiratory syndrome
PAM: porcine alveolar macrophages
RIN: RNA integrity number
TOM: topological overlap matrix
WGCNA: weighted gene co-expression network analysis

## References

[1] Rothschild M, Lunney J, Steibel J, Reecy J, Fritz E, Kerrigan M, Trible B, Rowland R (2011) Probing genetic control of swine responses to PRRSV infection: current progress of the PRRS host genetics consortium. BMC Proc 5(Suppl 4):S30 21645311 10.1186/1753-6561-5-S4-S30 PMC3108226

[2] Rowland RR, Lunney J, Dekkers J (2012) Control of porcine reproductive and respiratory syndrome (PRRS) through genetic improvements in disease resistance and tolerance. Front Genet 3:260 23403935 10.3389/fgene.2012.00260 PMC3565991

[3] Sun Y, Han M, Kim C, Calvert JG, Yoo D (2012) Interplay between interferon-mediated innate immunity and porcine reproductive and respiratory syndrome virus. Viruses 4:424–446 22590680 10.3390/v4040424 PMC3347317

[4] Miller L, Laegreid W, Bono J, Chitko-McKown C, Fox J (2004) Interferon type I response in porcine reproductive and respiratory syndrome virus-infected MARC-145 cells. Arch Virol 149:2453–2463 15338318 10.1007/s00705-004-0377-9 PMC7087254

[5] Rodríguez-Gómez I, Gómez-Laguna J, Barranco I, Pallarés F, Ramis G, Salguero F, Carrasco L (2013) Downregulation of antigen-presenting cells in tonsil and lymph nodes of porcine reproductive and respiratory syndrome virus-infected pigs. Transbound Emerg Dis 60:425–437 22816521 10.1111/j.1865-1682.2012.01363.x

[6] Inoue R, Tsukahara T, Sunaba C, Itoh M, Ushida K (2007) Simple and rapid detection of the porcine reproductive and respiratory syndrome virus from pig whole blood using filter paper. J Virol Methods 141:102–106 17188757 10.1016/j.jviromet.2006.11.030

[7] Hickey MJ, Kubes P (2009) Intravascular immunity: the host–pathogen encounter in blood vessels. Nat Rev Immunol 9:364–375 19390567 10.1038/nri2532

[8] Kommadath A, Bao H, Choi I, Reecy JM, Koltes JE, Fritz-Waters E, Eisley CJ, Grant JR, Rowland RR, Tuggle CK, Dekkers JCM, Lunney JK, Guan LL, Stothard P, Plastow GS (2017) Genetic architecture of gene expression underlying variation in host response to porcine reproductive and respiratory syndrome virus infection. Sci Rep 7:46203 28393889 10.1038/srep46203 PMC5385538

[9] Schroyen M, Eisley C, Koltes JE, Fritz-Waters E, Choi I, Plastow GS, Guan L, Stothard P, Bao H, Kommadath A, Reecy JM, Lunney JK, Rowland RRR, Dekkers JCM, Tuggle CK (2016) Bioinformatic analyses in early host response to porcine reproductive and respiratory syndrome virus (PRRSV) reveals pathway differences between pigs with alternate genotypes for a major host response QTL. BMC Genom 17:196 10.1186/s12864-016-2547-z PMC4782518 26951612

[10] Wilkinson JM, Ladinig A, Bao H, Kommadath A, Stothard P, Lunney JK, Harding JC, Plastow GS (2016) Differences in whole blood gene expression associated with infection time-course and extent of fetal mortality in a reproductive model of type 2 porcine reproductive and respiratory syndrome virus (PRRSV) infection. PLoS One 11:e0153615 27093427 10.1371/journal.pone.0153615 PMC4836665

[11] Betsou F, Gaignaux A, Ammerlaan W, Norris PJ, Stone M (2019) Biospecimen science of blood for peripheral blood mononuclear cell (PBMC) functional applications. Curr Pathobiol Rep 7:17–27

[12] Lim B, Kim S, Lim K-S, Jeong C-G, Kim S-C, Lee S-M, Park C-K, Te Pas MF, Gho H, Kim T-H, Lee K-T, Kim W-I, Kim J-M (2020) Integrated time-serial transcriptome networks reveal common innate and tissue-specific adaptive immune responses to PRRSV infection. Vet Res 51:128 33050948 10.1186/s13567-020-00850-5 PMC7552595

[13] Lim K-S, Cheng J, Putz A, Dong Q, Bai X, Beiki H, Tuggle CK, Dyck MK, Canada PG, Fortin F, Harding JCS, Plastow GS, Dekkers JCM (2021) Quantitative analysis of the blood transcriptome of young healthy pigs and its relationship with subsequent disease resilience. BMC Genom 22:614 10.1186/s12864-021-07912-8 PMC8361860 34384354

[14] Aran D, Hu Z, Butte AJ (2017) xCell: digitally portraying the tissue cellular heterogeneity landscape. Genome Biol 18:220 29141660 10.1186/s13059-017-1349-1 PMC5688663

[15] Jeong C-G, Khatun A, Nazki S, Lee S-I, Kim T-H, Kim K-S, Park C-K, Kim W-I (2019) Production and evaluation of PRRS resistant pigs. Korean J Vet Serv 42:1–7

[16] Khatun A, Nazki S, Jeong C-G, Gu S, Mattoo S, Lee S-I, Yang M-S, Lim B, Kim K-S, Kim B, Lee K-T, Park C-K, Lee S-M, Kim W-I (2020) Effect of polymorphisms in porcine guanylate-binding proteins on host resistance to PRRSV infection in experimentally challenged pigs. Vet Res 51:14 32075688 10.1186/s13567-020-00745-5 PMC7031929

[17] Nazki S, Khatun A, Jeong C-G, Mattoo S, Gu S, Lee S-I, Kim S-C, Park J-H, Yang M-S, Kim B, Park C-K, Lee S-M, Kim W-I (2020) Evaluation of local and systemic immune responses in pigs experimentally challenged with porcine reproductive and respiratory syndrome virus. Vet Res 51:66 32404209 10.1186/s13567-020-00789-7 PMC7222343

[18] Shabir N, Khatun A, Kim W-I (2014) Different immunological features of two genetically distinct type 2 porcine reproductive and respiratory syndrome (PRRS) viruses. Korean J Vet Serv 37:1–9

[19] Sun D, Khatun A, Kim W-I, Cooper V, Cho Y-I, Wang C, Choi E-J, Yoon K-J (2016) Attempts to enhance cross-protection against porcine reproductive and respiratory syndrome viruses using chimeric viruses containing structural genes from two antigenically distinct strains. Vaccine 34:4335–4342 27406935 10.1016/j.vaccine.2016.06.069

[20] Trapnell BC, Whitsett JA (2002) Gm-CSF regulates pulmonary surfactant homeostasis and alveolar macrophage-mediated innate host defense. Annu Rev Physiol 64:775–802 11826288 10.1146/annurev.physiol.64.090601.113847

[21] Guo Z, Zhang Z, Prajapati M, Li Y (2021) Lymphopenia caused by virus infections and the mechanisms beyond. Viruses 13:1876 34578457 10.3390/v13091876 PMC8473169

[22] Taylor GS, Long HM, Brooks JM, Rickinson AB, Hislop AD (2015) The immunology of Epstein-Barr virus–induced disease. Annu Rev Immunol 33:787–821 25706097 10.1146/annurev-immunol-032414-112326

[23] Wu Q, Han Y, Wu X, Wang Y, Su Q, Shen Y, Guan K, Michal JJ, Jiang Z, Liu B, Zhou X (2022) Integrated time-series transcriptomic and metabolomic analyses reveal different inflammatory and adaptive immune responses contributing to host resistance to PRRSV. Front Immunol 13:960709 36341362 10.3389/fimmu.2022.960709 PMC9631489

[24] Deng G, Zhou G, Zhang R, Zhai Y, Zhao W, Yan Z, Deng C, Yuan X, Xu B, Dong X, Zhang X, Zhang X, Yao Z, Shen Y, Qiang B, Wang Y, He F (2008) Regulatory polymorphisms in the promoter of CXCL10 gene and disease progression in male hepatitis B virus carriers. Gastroenterology 134:716–726 18325387 10.1053/j.gastro.2007.12.044

[25] Schneider WM, Chevillotte MD, Rice CM (2014) Interferon-stimulated genes: a complex web of host defenses. Annu Rev Immunol 32:513–545 24555472 10.1146/annurev-immunol-032713-120231 PMC4313732

[26] Lim B, Kim S-C, Kim W-I, Kim J-M (2023) Integrative time-serial networks for genome-wide lncRNA-mRNA interactions reveal interferon-inducible antiviral and T-cell receptor regulations against PRRSV infection. Dev Comp Immunol 147:104759 37315774 10.1016/j.dci.2023.104759

[27] Yang L, Zhang Y-J (2017) Antagonizing cytokine-mediated JAK-STAT signaling by porcine reproductive and respiratory syndrome virus. Vet Microbiol 209:57–65 28069291 10.1016/j.vetmic.2016.12.036 PMC7117332

[28] Chen Z, Laurence A, Kanno Y, Pacher-Zavisin M, Zhu B-M, Tato C, Yoshimura A, Hennighausen L, O’Shea JJ (2006) Selective regulatory function of Socs3 in the formation of IL-17-secreting T cells. Proc Natl Acad Sci 103:8137–8142 16698929 10.1073/pnas.0600666103 PMC1459629

[29] O’Shea JJ, Plenge R (2012) JAK and STAT signaling molecules in immunoregulation and immune-mediated disease. Immunity 36:542–550 22520847 10.1016/j.immuni.2012.03.014 PMC3499974

[30] Ikeda M, Mori K, Ariumi Y, Dansako H, Kato N (2009) Oncostatin M synergistically inhibits HCV RNA replication in combination with interferon-α. FEBS lett 583:1434–1438 19332062 10.1016/j.febslet.2009.03.054

[31] Larrea E, Aldabe R, Gonzalez I, Segura V, Sarobe P, Echeverria I, Prieto J (2009) Oncostatin M enhances the antiviral effects of type I interferon and activates immunostimulatory functions in liver epithelial cells. J Virol 83:3298–3311 19158240 10.1128/JVI.02167-08 PMC2655580

[32] Yang L, Wang R, Ma Z, Xiao Y, Nan Y, Wang Y, Lin S, Zhang Y-J (2017) Porcine reproductive and respiratory syndrome virus antagonizes JAK/STAT3 signaling via nsp5, which induces STAT3 degradation. J Virol 91:e02087-16 27881658 10.1128/JVI.02087-16 PMC5244345

[33] Carson JL, Stanworth SJ, Roubinian N, Fergusson DA, Triulzi D, Doree C, Hebert PC (2016) Transfusion thresholds and other strategies for guiding allogeneic red blood cell transfusion. Cochrane Database Syst Rev 10:CD002042 27731885 10.1002/14651858.CD002042.pub4 PMC6457993

[34] Jones MJ, Goodman SJ, Kobor MS (2015) DNA methylation and healthy human aging. Aging Cell 14:924–932 25913071 10.1111/acel.12349 PMC4693469

[35] Kang S, Brown HM, Hwang S (2018) Direct antiviral mechanisms of interferon-gamma. Immune Netw 18:e33 30402328 10.4110/in.2018.18.e33 PMC6215902

[36] Glass WG, Rosenberg HF, Murphy PM (2003) Chemokine regulation of inflammation during acute viral infection. Curr Opin Allergy Clin Immunol 3:467–473 14612671 10.1097/00130832-200312000-00008

[37] Grandvaux N, Servant MJ, tenOever B, Sen GC, Balachandran S, Barber GN, Lin R, Hiscott J (2002) Transcriptional profiling of interferon regulatory factor 3 target genes: direct involvement in the regulation of interferon-stimulated genes. J Virol 76:5532–5539 11991981 10.1128/JVI.76.11.5532-5539.2002 PMC137057

[38] Lagumdzic E, Pernold CP, Ertl R, Palmieri N, Stadler M, Sawyer S, Stas MR, Kreutzmann H, Rümenapf T, Ladinig A, Saalmüller A (2023) Gene expression of peripheral blood mononuclear cells and CD8+ T cells from gilts after PRRSV infection. Front Immunol 14:1159970 37409113 10.3389/fimmu.2023.1159970 PMC10318438

[39] Kaplanski G (2018) Interleukin-18: biological properties and role in disease pathogenesis. Immunol Rev 281:138–153 29247988 10.1111/imr.12616 PMC7165732

[40] Stanley ER, Chitu V (2014) CSF-1 receptor signaling in myeloid cells. Cold Spring Harbor Perspect Biol 6:a021857 10.1101/cshperspect.a021857 PMC4031967 24890514

