# Supplementary figures and images for: Cell deconvolution-based integrated time-series network of whole blood transcriptome reveals systemic antiviral activities and cell-specific immunological changes against PRRSV infection
Source: Vet Res. 2025 Jan 22;56:19. doi: 10.1186/s13567-025-01451-w (PMC11755918; doi:10.1186/s13567-025-01451-w)

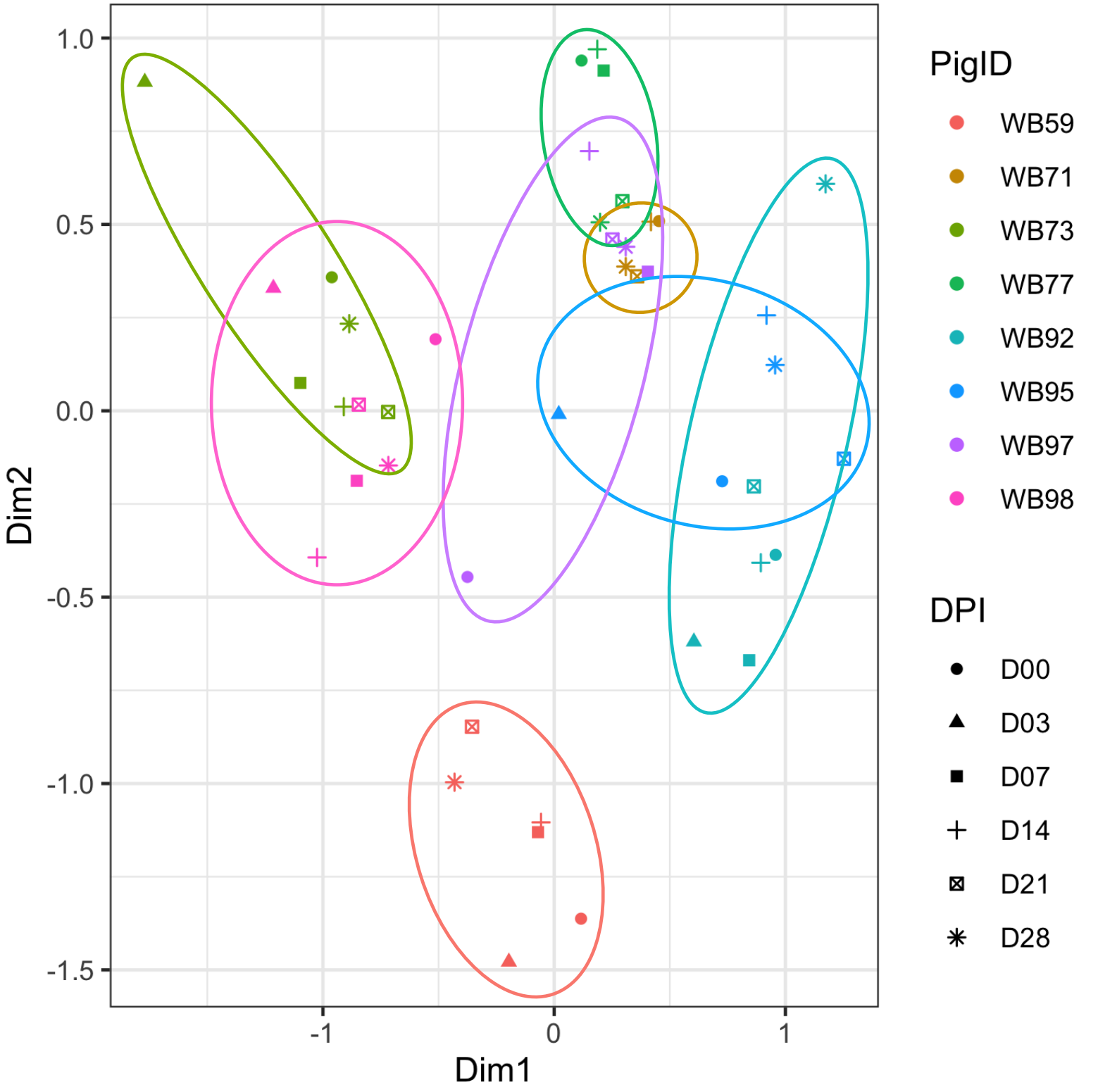

Supplement: Supplementary file 2 — Additional file 2. Multidimensional scaling (MDS) based on whole blood transcriptomes in PRRSV infection. Individual effects were found to be greater than temporal effects. These features were considered and adjusted while profiling the differentially expressed genes. [file 13567_2025_1451_MOESM2_ESM.pdf]

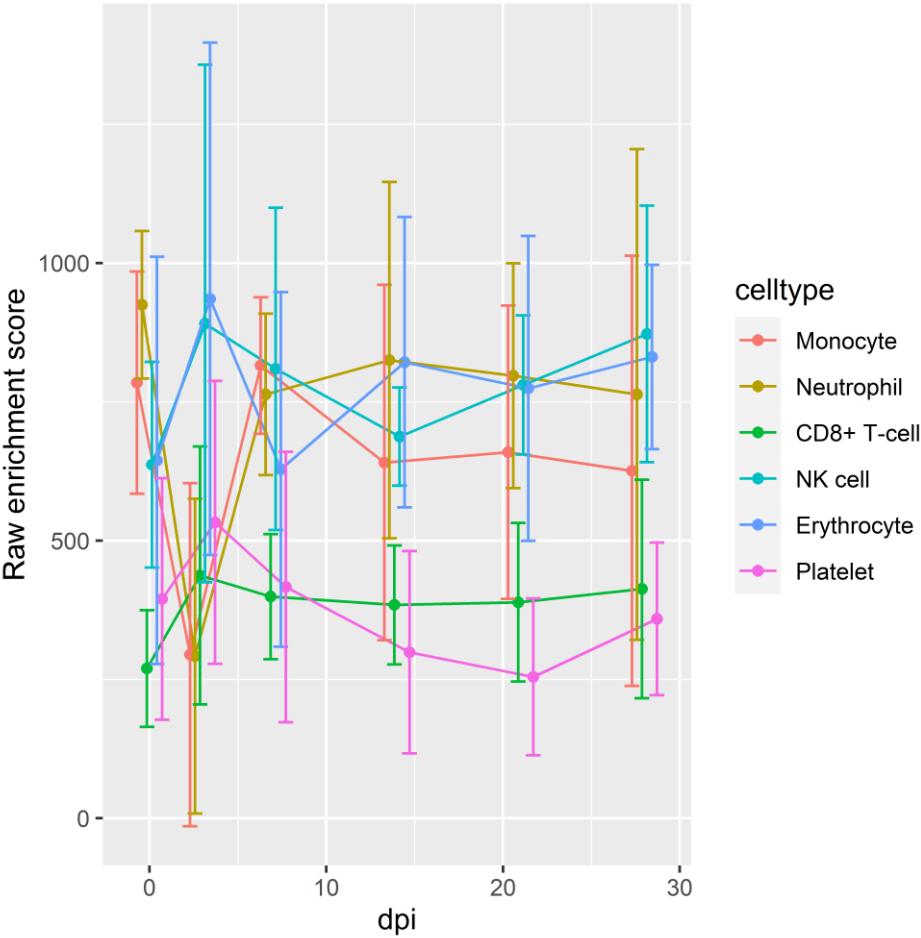

Supplement: Supplementary file 6 — Additional file 6. Changes in raw enrichment scores for representative cell types. [file 13567_2025_1451_MOESM6_ESM.pdf]
